# Expression of interleukin-18 in primary Sjögren syndrome and its potential mechanisms with disease: A systematic review and meta-analysis

**DOI:** 10.1097/MD.0000000000041919

**Published:** 2025-03-21

**Authors:** Ying Deng, Yijue Wang, Yijuan Cheng, Min Lei, Yingyu Luo, Wenling Gu, Boyu Cai, Li Li, Nannan Zhang

**Affiliations:** a National Center for Birth Defect Monitoring, Key Laboratory of Birth Defects and Related Diseases of Women and Children, Ministry of Education, West China Second University Hospital, Sichuan University, Chengdu, China; b West China Hospital of Stomatology, Sichuan University, Chengdu, China; c West China Hospital, Sichuan University, Chengdu, China; d Division of Renal and Endocrinology, Qin Huang Hospital, Xi’an, China.

**Keywords:** interleukin-18, meta-analysis, primary Sjögren syndrome

## Abstract

**Background:**

Interleukin-18 (IL-18), an IL-1 family cytokine with potent ability to induce interferon gamma production and enhance Th1 response, was elevated in a group of autoimmune diseases including primary Sjögren syndrome (pSS). IL-18 dysregulation can contribute to the pathogenesis of these disorders by exerting function in innate and adaptive immunity. This study is aimed at comparing the level of IL-18 in pSS patients and explored the association between IL-18 and pSS.

**Methods:**

Six databases, including PubMed, Web of Science, Embase, Ovid Medline, Scopus and China National Knowledge Infrastructure, were searched. The quality of the included studies was assessed using the Newcastle–Ottawa Scale criteria. We analyzed IL-18 concentrations in the serum and tear of pSS patients and healthy controls (HCs), and review the potential mechanism between IL-18 and pSS.

**Results:**

Eleven articles were included in this study, which reported IL-18 levels in serum and tear of pSS patients and HCs. IL-18 levels in pSS patients were significantly higher than those in HCs (standard mean difference = 1.28, 95% confidence interval 0.75–1.82, *P* < .001), with substantial heterogeneity (*I*^2^ = 90%, *P* < .001) among the studies. The level of IL-18 in serum was analyzed separately and was also higher than those in HCs (standard mean difference = 1.28, 95% confidence interval 0.68–1.88, *P* < .001), with significant heterogeneity (*I*^2^ = 91%, *P* < .001). Apart from that, the potential pathogenesis of pSS was concluded comprehensively.

**Conclusion:**

IL-18 abundance was evidently elevated in pSS patients and can thus likely be used as a reliable biomarker to monitor and track the progression of pSS, and further to become a good target for treatment of pSS patient.

## 1. Introduction

Sjögren syndrome (SS) is a systemic chronic autoimmune disease characterized by ocular and oral dryness resulting from lacrimal and salivary gland dysfunction, with a frequency in general population ranging from 0.01 to 0.72%.^[1,2]^ By virtue of the decreasing salivary secretion, some serious oral clinical manifestations such as rampant caries, burning sensation of the oral mucosa and lingual papilla atrophy may appear while the disease progresses.^[3,4]^ Besides, exocrine gland inflammation is also a defining feature of SS, which is usually measured by the focus score of lymphocytic infiltration and the level of inflammatory cytokines.^[5]^ This disease can present alone as primary Sjögren syndrome (pSS) or associated with other systemic autoimmune diseases as secondary Sjögren syndrome.^[1]^ Moreover, the patient with pSS suffers a higher risk of lymphoma (mainly B cell non-Hodgkin lymphomas) and cardiovascular diseases compared to that in the general population.^[6]^

Although the underlying cause of pSS and pathogenesis remain obscure, important efforts have been made during the past few years. Environmental, genetic, epigenetic, and hormonal factors are implicated in pSS pathogenesis.^[7]^ In essence, the pathogenesis of pSS is a result of a complex interaction between the activated immune system (including the innate immunity and acquired immunity).^[8]^ The expression of 23 genes in the interferon (IFN) pathways was found to be different between pSS patients and controls. Among these genes, 21 genes including toll-like receptor 8 and toll-like receptor 9, 2 members of the toll-like receptor family, showed increased expression in pSS patients. Two genes (suppressor of cytokine signaling 3, and C-C motif chemokine ligand 18) had a significantly decreased expression. Most of these genes are involved in IFN-α and IFN-β pathways.^[9]^ Notably, helper T cell populations are thought to play an important role in pSS, and some scientists confirm that Th1 cells and Th17 cells initiate SS while Th2 cells and Follicular helper T cell predominate as the disease progresses.^[10]^ Additionally, either a reduced natural killer (NK) cell number or an impaired NK cell function were identified in patients, which might permit the persistence of abnormal autoimmune response.^[11]^

**Figure 1.**
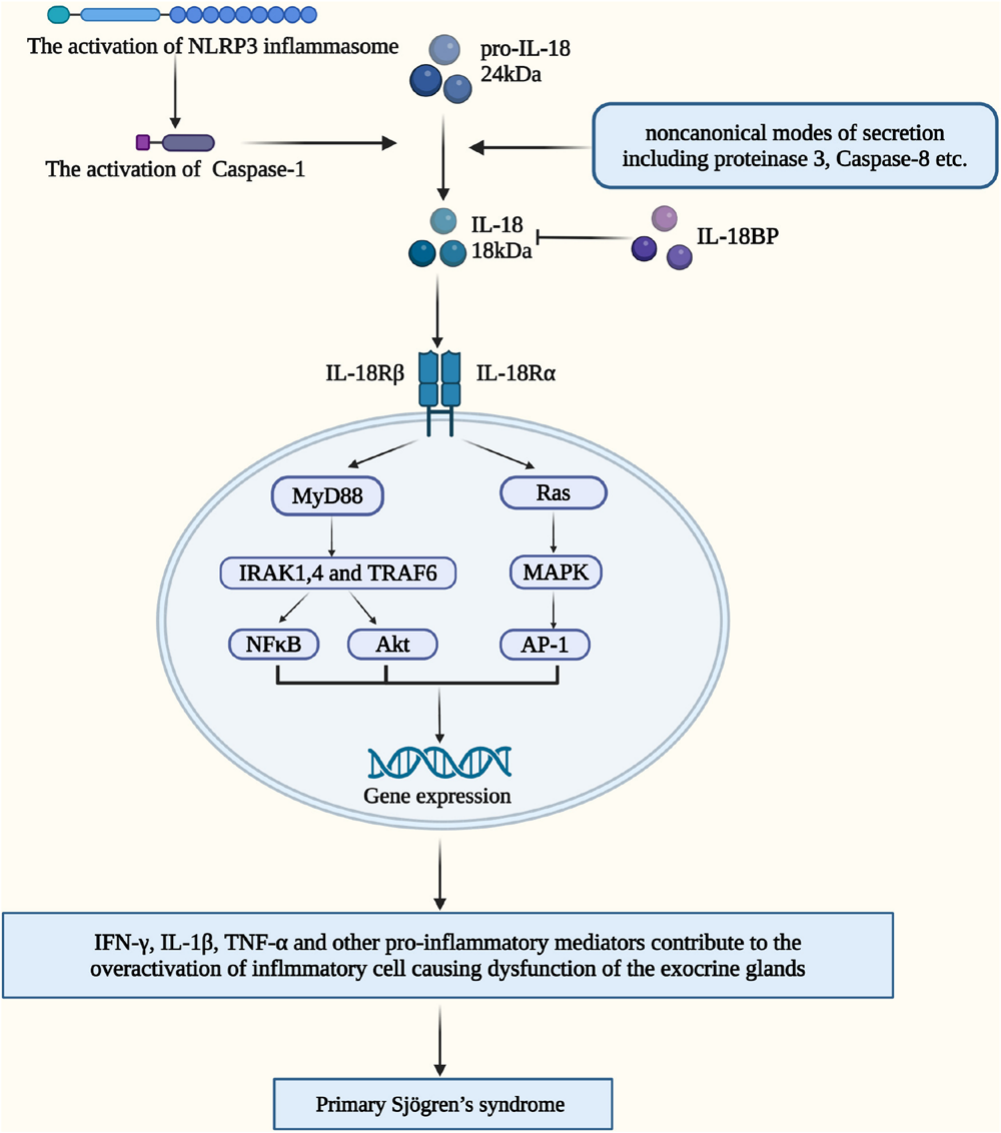
Interleukin-18 signaling pathway and its functional binding with IL-18 receptor. This figure shows both classical and nonclassical pathways of IL-18 activation, exploring its possible signaling pathways which may lead to primary Sjögren’s syndrome (figure created by Biorender).

Interleukin-18 (IL-18), a member of the interleukin-1 superfamily, can be produced by various cells types, including circulating monocytes, resident macrophages, and dendritic cells in its primary extracellular form.^[12]^ IL-18 works by binding to IL-18 receptor (IL-18R), which is found on a wide range of cells, including macrophages, monocytes neutrophils, NK cells, endothelial cells, and smooth muscle cells.^[13]^ By binding to IL-18R, IL-18 stimulates macrophages, monocytes neutrophils, NK cells, endothelial cells to produce pro-inflammation cytokines such as IFN-γ. IL-18/IL-18R complex could activate various intracellular signaling molecules such as Myeloid differentiation primary response protein 88, interleukin-1 receptor-associated kinase, and TNF receptor associated factor 6, and induce the activation of the NF-kB, and p38-MAPK signaling pathways (Fig. 1).^[12]^ IL-18 plays an important role in several inflammatory-based disease and autoimmune condition, such as systemic lupus erythematosus,^[14]^ psoriasis,^[15]^ and rheumatoid arthritis.^[16]^ Some scholars have found that there is a significant increasing trend of IL-18 in pSS patients, which indirectly indicates that IL-18 plays an important role in the pathogenesis of pSS.^[17,18]^ IL-18 has been shown to facilitate the production and maintenance of Th17 cells, and IL-18-neutralizing antibody could inhibit Th17 immune response in the psoriasis-like mouse model. Moreover, in the same mouse model, high mobility group box 1 protein-induced IL-18 expression and secretion were found to facilitate the formation of psoriasiform dermatitis.^[19]^ Taken together, these studies reveal that IL-18 may be involved in the pathogenesis of pSS by participating in the regulation of immune cells, and these biological activities on T-cell subsets has made IL-18 as a good target of the research in pSS.

Although lots of efforts have been made, no comprehensive study has analyzed potential associations between IL-18 expression and pSS. Therefore, the objectives of this study are to evaluate IL-18 levels in pSS patients and summarize the potential mechanisms in existing research.

Therefore, the purpose of this systematic review and meta-analysis was to clarify (i) alterations in serum and tear IL-18 protein levels of pSS patients; (ii) existing evidence between the IL-18 level and disease severity; (iii) potential regulatory mechanisms of IL-18 in the development of pSS.

## 2. Methods

### 2.1. Search strategy

The protocol was not registered in the International Prospective Register of Systematic Reviews (PROSPERO). Up to June 30, 2024, the search sources included PubMed, Web of Science, Embase, Ovid Medline, Scopus and China National Knowledge Infrastructure. We used the keywords (“Interleukin-18” OR “IFN-gamma-inducing factor” OR “IL-18” OR “Interferon-gamma-inducing factor”) and (“Sjögren syndrome” OR “Sicca syndrome”). The searches were conducted by 2 independent researchers (Yijue Wang and Yijuan Cheng), and any discrepancies were resolved through discussion.

### 2.2. Selection criteria

The following criteria were used to choose participants: (i) original clinical trials reporting serum and tear IL-18 levels detected by enzyme-linked immunosorbent assay of patients with pSS, healthy controls (HCs); (ii) patients diagnosed as pSS based on standard diagnostic criteria such as the American College of Rheumatology criteria or European League Against Rheumatism (EULAR) criteria for pSS; and (iii) mean and standard deviation (SD) of IL-18 levels were reported or calculated from the original data or obtained by contacting the authors. The following were the exclusion criteria: (i) secondary Sjögren syndrome patients and HCs suffering from other systemic autoimmune diseases; and (ii) nonclinical research in animal models or cell lines. Ying Deng accomplished the composition of the article. Two researchers (Yijue Wang and Yijuan Cheng) independently screened the titles and abstracts of all retrieved studies and conducted a full-text evaluation for future screening based on the eligibility criteria. Min Lei was responsible for data entry. Any discrepancies between the findings of the 2 researchers were resolved through discussion, and if no consensus was reached, a senior researcher (Nannan Zhang) helped in the decision-making.

### 2.3. Data extraction

We extracted the following information from all studies included: author names, publication years, country, sample sizes, sample types, patient age and sex, disease duration (years), mean and SD of IL-18 concentrations (pg/mL), serological parameters [e.g., serum levels of total immunoglobulins, IgA, IgG, IgM, C3, and C4 (g/L)], and clinical parameters [e.g., EULAR Sjögren syndrome disease activity index score, EULAR Sjögren syndrome patient reported index score, and Sjögren syndrome disease activity index score]. However, most of the studies lack that data in the healthy control group. The correlation coefficient (*r*) with IL-18 was also not recorded.

### 2.4. Quality assessment

The primary studies were subjected to the quality assessment of Newcastle–Ottawa quality assessment scale by 2 investigators. The overall quality score varied from 0 to 9, based on an assumption of equal weight for all elements included in the quality assessment scale, and items included the assessment of patient selection, group comparability, and the quality of the collection process/cytokine assay. In this meta-analysis, studies with values of 7 to 9 were considered generally high quality, studies with scores of 4 to 6 were considered moderate quality, and studies with scores of 1 to 3 were considered relatively low quality. Data extraction and quality assessment of eligible studies were performed independently by 2 reviewers (Yijue Wang and Yijuan Cheng). Any disagreement was resolved through discussion or arbitrated by a third reviewer (Nannan Zhang).

### 2.5. Statistical analysis

RevMan 5.4 and State 15.0 software was used for statistical analysis. *To investigate significant changes in IL-18 levels between pSS patients and controls, the sample size and mean SD were used to calculate the standard mean difference (SMD) with 95% confidence interval (CI).* Among the 11 selected studies, 6 studies provided mean and SD. The other 5 studies which only provided values of median ± interquartile range or median ± range were converted into the right form using a standard method.^[20]^ A random-effects model was used to pool SMD values. Heterogeneity among studies was assessed using the Cochran *Q* test and I2 statistical parameter, and values of *P* < .1 or *I*^2^ > 50% represented substantial heterogeneity. Sources of heterogeneity were studied by meta-regression if high heterogeneity was found in the comparisons including more than 10 studies, and these sources were considered if *P* < .05.

A funnel plot was used to visualize the publication bias of the included literature, and the degree of asymmetry was quantified using Egger test if statistical significance was detected. The results of the Egger test (*P* > .05) were considered to have no significant publication bias.

## 3. Results

### 3.1. Study characteristics

**Figure 2.**
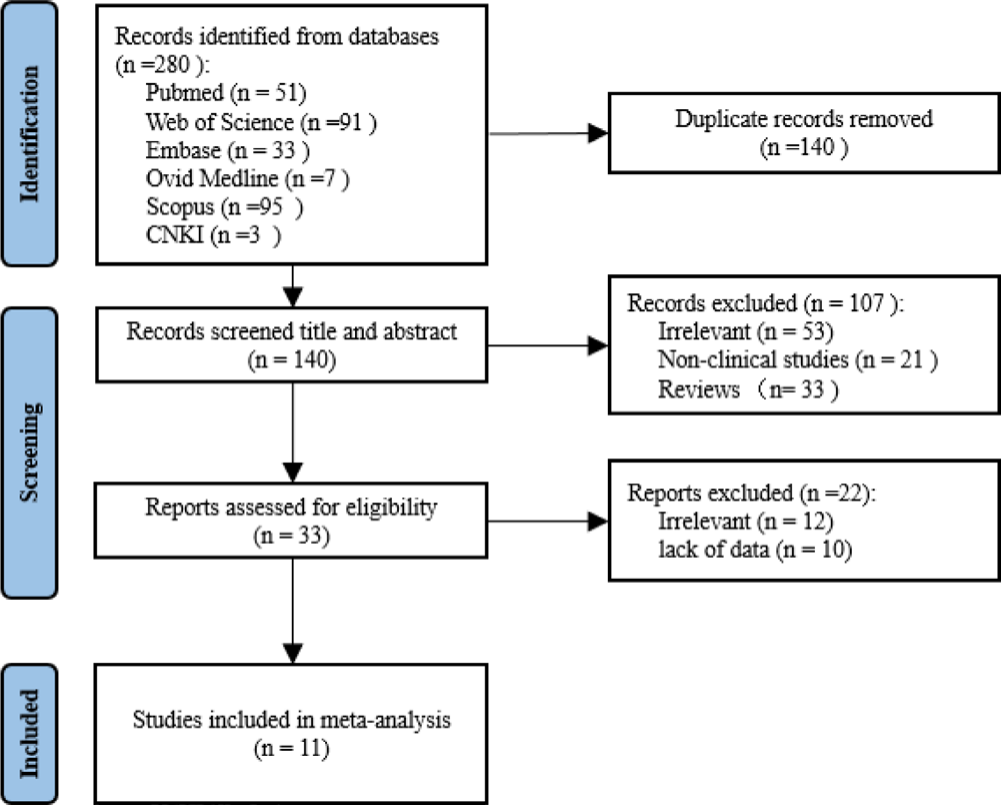
Flow diagram of study selection in the meta-analysis. Two hundred and eighty records are retrieved from 6 databases according to our search strategy. Hundred and forty duplicated records are excluded. Hundred and seven records are excluded after our primary screening, including those irrelevant to the theme (n = 53), and the nonclinical studies like animal experiments and bioinformatics analysis (n = 21) and review and systematic reviews (n = 33). Furtherly, 22 records are excluded after the secondary screening, including those irrelevant to the theme (n = 12) and those unavailable for concrete data (n = 10). Finally, 11 records are included in the meta-analysis. CNKI = China National Knowledge Infrastructure, HCs = healthy controls.

According to the search strategy, 280 records were retrieved from 6 databases, and 140 records were excluded due to duplication. We then excluded 107 records including irrelevant studies, nonhuman studies, and reviews from the remaining 140 records after screening the titles and abstracts. Full-text assessment was conducted among the remaining 33 records, 12 records were excluded for being irrelevant, and 10 records were excluded due to lack of data. Finally, 11 articles were included in this systematic review and meta-analysis, including 10 in English and 1 in Chinese. The selecting process is record in Figure 2. Six articles reported a correlation between IL-18 levels in the serum of pSS patients and their serological or clinical parameters. Five articles only reported the IL-18 levels in pSS and controls. Among the 11 articles, IL-18 levels in the mean ± SD format were extracted directly from 6 articles. The other 5 articles only reported the median, interquartile range, or range of IL-18 levels, and we transformed the data into mean and SD values. According to the quality assessment criteria above, 6 articles were of high quality and 5 articles were of moderate quality indicating that this meta-analysis was based on high quality evidence (see Table S1, Supplemental Digital Content, http://links.lww.com/MD/O538 which illustrates this meta-analysis was based on high quality evidence).

### 3.2. Systematic review of IL-18 and pSS

Most articles suggest that there is a significant correlation between pSS-related disease parameters and IL-18 level. Yan Chen study compared IL-18 level in mild disease group and active disease group and concluded that the latter had significantly higher IL-18 levels.^[17]^ The same conclusion was mentioned in Wang Liuqing study.^[18]^ In Seong-Kyu Kim study, Sjögren syndrome disease activity index score was noted to be related with the mRNA expression level of IL-18 (*R* = 0.36, *P* = .04).^[21]^ Besides, the positive correlation between serological parameters and IL-18 level was also observed. Wang Liuqing study discovered that total IL-18 serum levels were positively related to IgG levels.^[18]^ However, a limited number of studies were lack of those data (Table 1, which recorded all relevant data that indicates the severity of the disease).

**Table 1:** Characteristics of studies included in the systematic review.

			Case			Control	
Author and year (Ref)	Country	Study design	Serological parameters*	Clinical parameters†	Duration years	Serological parameters	Quality assessment‡
Peter Olsson 2018	Sweden	Case-control	Median (IQR): IgG: 13.0 (10.1, 15.5); C3: 1.01 (0.86, 1.20); C4: 0.18 (0.13, 0.21)	Median (IQR): ESSPRI score: 6 (5, 7); ESSDAI score: 7 (1, 10)	Median (IQR): 12 (6, 18)	NA	High
Yan Chen 2016	China	Cross-sectional	C3↓ (%): 42.9; C4↓ (%): 40; IgG↑ (%): 60; IgA↑ (%): 8.6; IgM↑ (%): 8.6	Mean (SD): ESSDAI score: 9.3 (6.8)	Mean (SD): 2.71 (3.18)	NA	High
Liangliang Niu 2015	China	Cross-sectional	NA	NA	NA	NA	Middle
Serena Colafrancesco 2012	Italy	Cross-sectional	NA	NA	NA	NA	Middle
Toshiyuki Aramaki 2009	Japan	Cross-sectional	NA	NA	NA	NA	Middle
P. Epiksson 2004	Sweden	Cross-sectional	Median (range): IgG1: 16.1 (4.7–44.3); IgA: 3.4 (0.0–8.4); IgM: 1.5 (0.7–2.4)	NA	NA	Median (range): IgG1: 6.0 (3.8–8.6); IgA: 2.1 (1.1–3.6); IgM: 1.2 (0.54–1.9)	High
Michele Bombardieri 2004	UK	Case-control	IgG↑ (%): 16; IgA↑ (%): 59	NA	Median (range): 5.93 (0.17–30)	NA	High
Chai Kexia 2011	China	Cross-sectional	NA	NA	NA	NA	High
Yasumori Izumi 2006	Japan	Cross-sectional	NA	NA	NA	NA	Middle
Wang Liuqing 2017	China	Cross-sectional	Mean (SD): IgG: 18.4 (22.7)	NA	Mean (SD): 6.3 (4.7)	NA	Middle
Seong-Kyu Kim 2017	Korea		Median (IQR): C3: 1.03 (0.89–1.22); C4: 0.22 (0.18–0.25)	Median (IQR): ESSDAI score: 2.0 (0.0–4.0); SSDDI score: 1.0 (0.0–2.0)	Median (IQR): 4.0 (2.0–8.0)		High

* Serological parameters: serum levels of total immunoglobulins, IgA, IgG, IgM, C3, and C4 (g/L).

† Clinical parameters: EULAR Sjögren syndrome disease activity index (ESSDAI) score, EULAR Sjögren syndrome patient reported index (ESSPRI) score, and Sjögren syndrome disease damage index (SSDDI) score.

‡ Quality assessment was conducted using the nine-star Newcastle–Ottawa Scale (NOS), studies with NOS scores ≥ 6 were high quality, studies with NOS scores < 6 and NOS scores ≥ 3 were relatively middle quality, and studies with NOS scores < 3 were low quality following a standard method.

### 3.3. Meta-analysis of IL-18 levels in pSS vs controls

**Figure 3.**
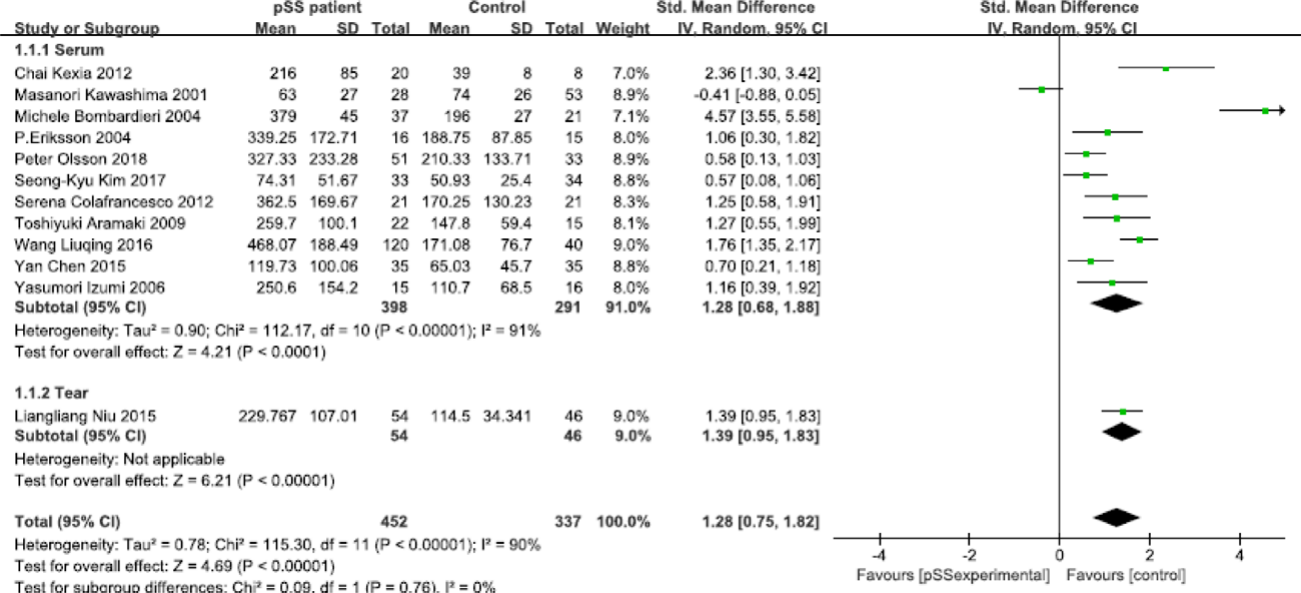
Comparison of IL-18 concentrations from serum and tear between primary Sjögren’s syndrome (pSS).

All included studies reported elevated IL-18 levels in pSS patients. Eleven articles reported IL-18 levels in pSS patients and HCs, including 708 participants. Among these studies, 10 articles reported IL-18 levels in serum,^[11,17,18,21–27]^ and 1 reported IL-18 levels in tear.^[28]^ IL-18 levels in pSS patients were significantly higher than those in HCs, (SMD = 1.28, 95% CI 0.75–1.82, *P* < .001), with substantial heterogeneity (*I*^2^ = 90%, *P* < .001) among the studies. Subgroup analysis subsequently showed increased IL-18 in serum of pSS patient (SMD = 1.28, 95% CI 0.68–1.88, *P* < .001), with significant heterogeneity (*I*^2^ = 91%, *P* < .001) (Fig. 3).

### 3.4. Publication bias

We used funnel plots and Egger test to assess publication bias of each comparison with statistical significance in the meta-analysis. No significant publication bias was found in the case of comparison of IL-18 levels between pSS patients and HCs, indicating by the asymmetry of the funnel plot (see Figure S1, Supplemental Digital Content, http://links.lww.com/MD/O539 which illustrates the asymmetry of the funnel plot). and the results of Egger test (*P* = .12). Therefore, the results of this meta-analysis are valid.

## 4. Discussion

Although the pathophysiology of pSS is still unknown, previous studies have shown that cytokines may regulate inflammation and immunopathology in pSS patients by interacting with inflammatory cells. We discovered that cytokine IL-18 has a considerable propensity to rise in pSS patients. Even though many researchers have discovered elevated IL-18 expression in pSS patients’ serum and tear, to our knowledge, no systematic investigation has compiled these findings. In this study, IL-18 levels in the blood of pSS patients were significantly higher than those in control group. The same results had not been observed in tear or salivary of pSS patients; the possible reason may be the small sample size in these studies.

The pooled results showed that IL-18 levels were robustly increased in the serum of pSS patients as compared to those in both types of controls in this meta-analysis. After the process of systematic review, IL-18 levels had a positive correlation with both serological and clinical parameters in pSS patients. Similarly, J. Loureiro-Amigo study also found that EULAR Sjögren syndrome disease activity index score was associated with IL-18 level using the multiplex immunoassay.^[29]^ Based on the above results, IL-18 may play a pivotal role in the development of pSS symptoms and may reflect disease severity to a certain extent. At the gene level, previous studies showed a significant increase of IL-18 gene expression in patients with pSS.^[30]^ Apart from that, IL-18 activated T-cell subsets and other immune cells, which has also made it an attractive target for pSS treatment.

Interleukin-18 (IL-18) is a member of IL-1 superfamily. The main extracellular form of IL-18 is all synthesized as biologically inactive precursor molecules inside cells, producing by circulating monocytes, resident macrophages, and dendritic cells.^[12]^ To be secreted and matured, it requires proteolytic processing, relied on the action of caspase-1, which is a curial component of NLRP3 (NOD-like receptor thermal protein domain associated protein 3) inflammasome.^[31,32]^ It is also demonstrated that the secretion of IL-18 is increased by the action of P2X7 (recombinant purinergic receptor P2X) receptor–NLRP3 inflammasome complex.^[33]^ In addition, pro-IL-18 was secreted as an inactive protein and processed by Caspase-1 into an active form, and IL-18 was also secreted by noncanonical modes by proteinase 3 directly stimulation.^[34]^ And Fas activated Caspase-8 in macrophages and dendritic cells, promoting IL-18 production.^[35]^

Previous reviews have elucidated the abnormal secretion of IL-18 and IL-1β due to activation of NLPR3 inflammasome, significantly increasing disease severity and lymphoma risk in pSS patients.^[21,36]^ Further studies also should focus on the downstream regulation mechanisms of inflammation after IL-18 activation. Meanwhile, the specific content changes and mechanism of IL-18 binding protein as a natural antagonist in pSS patients need to be more explored.

Several studies have revealed the potential immunity regulation function of IL-18 in pSS patients. IL-18 influences the function of helper T cell populations, particularly Th1 cells. The primary role of IL-18 is to induce IFN-γ secretion from Th1 cells. IFN-γ, in conjunction with IL-12, promotes Th1 cells differentiation. In the period of initiation, pSS is a Th1-dominated disease, leading to the high levels of IFN-γ and its related cytokines (IFNα/β and IL-12) in pSS patients.^[9]^ IL-18 is also able to work synergistically with IL-12 to activate Th2 cells, Th17 cells, NK cell, etc^[37]^ and produce high levels of autoantibodies (including RF, IgG, IgA, anti-Ro and anti-La, etc)^[38,39]^ and the fibrosis of exocrine glandular tissue.^[39,40]^ Simultaneously, IL-18 induced the overexpression of IL-17 in pSS patients,^[41–43]^ which may be considered as an additional inducer of autoantibodies.

Besides, research observed that patients with pSS have impaired NK cell function accompanied by increasing IL-18 concentration.^[26]^ Although the recent study has shown that IL-18 can activate NK cells,^[12]^ the potential regulatory mechanisms between IL-18 and NK cells are need to be more explored. Mature IL-18 critically involved in the salivary gland inflammation and has a role in modulating the inflammatory response in primary Sjogren syndrome. IL-18 could represent an interesting biomarker for lymphomagenesis in pSS, opening the novel opportunities for the early diagnosis of lymphoproliferative complications and the development of potential targeted therapies for pSS.

Demyelinating diseases are a group of autoimmune disorders caused by the demyelination of the nervous system. The studies on demyelinating diseases suggest that downregulating the immune system and inducing remyelination are very promising therapeutic approaches.^[44,45]^ Both demyelinating diseases and pSS are autoimmune disorders, the future therapeutic focuses for demyelinating diseases could also provide new insights for the treatment of Sjögren syndrome.

In conclusion, IL-18-mediated T cell response may hold an implicated role in pSS, and the inhibition of IL-18 can be a potential therapy for pSS. IL-18-mediated pathology in the development of pSS should be further studied.

Our study has its strengths and limitations. We have collected and included articles published over the past 2 decades related to IL-18 and pSS, which make our article comprehensive. In this systematic review and meta-analysis, we used various biomarkers to evaluate the disease progression and severity in the included studies, and provided a broader insight on the possible association between IL-18 and pSS. However, the study also has certain limitations. The heterogeneity between studies is 1 main limitation, which is related to the different geographical location, small sample size, year of publication, and disease duration. Besides, the inconsistencies in the sensitivity of detection methods and detection reagents can be an important factor causing heterogeneity. We didn’t also analyze the correlation between IL-18 levels and disease parameters in pSS patients, as the lack of related data in these studies.

## 5. Conclusion

The study reveals a strong association between illness severity and IL-18 levels, and further highlights the need for additional research to explore the underlying mechanisms. Quantitative analysis showed that maternal IL-18 levels were significantly increased in the serum of pSS patient. High IL-18 status may be associated with the pathogenesis of pSS. However, the interpretive caution is necessitated due to the limited quantity of studies and their substantial heterogeneity.

## 6. Strengths and limitations of this study

A comprehensive review and meta-analysis included the most published studies related to IL-18 and pSS, and provide the evidence of a strong association between illness severity and IL-18 levels.

IL-18 might be a reliable biomarker to monitor and track the progression of pSS.

The interpretation of the results was affected by the sample sizes of the included studies.

Heterogeneity was observed in the comparisons.

## Author contributions

**Data curation:** Yijuan Cheng, Min Lei, Li Li.

**Formal analysis:** Ying Deng.

**Investigation:** Yijue Wang, Yijuan Cheng, Wenling Gu, Boyu Cai.

**Methodology:** Nannan Zhang.

**Validation:** Yingyu Luo.

**Writing – review & editing:** Ying Deng.

## Supplementary Material

SUPPLEMENTARY MATERIAL
