# Supplementary material for: Expression of interleukin-18 in primary Sjögren syndrome and its potential mechanisms with disease: A systematic review and meta-analysis
Source: Medicine (Baltimore). 2025 Mar 21;104(12):e41919. doi: 10.1097/MD.0000000000041919 (PMC11936574; doi:10.1097/MD.0000000000041919)
Supplement: SUPPLEMENTARY MATERIAL [file medi-104-e41919-s001.docx]

| Study | Year | Selection | Comparability | Exposure | Quality score |  |
| --- | --- | --- | --- | --- | --- | --- |
| Peter Olsson | 2018 | **** | * | ** | 7 | |
| Yan Chen | 2015 | *** | ** | ** | 8 | |
| Liangliang Niu | 2015 | ** | * | *** | 6 | |
| Serena Colafrancesco | 2012 | ** |  | *** | 5 | |
| Toshiyuki Aramaki | 2009 | ** | * | *** | 6 | |
| P. Epiksson | 2004 | **** | ** | *** | 9 | |
| Michele Bombardieri | 2004 | *** | * | *** | 7 | |
| Chai Kexia | 2012 | *** | ** | *** | 8 | |
| Yasumori Izumi | 2006 | ** | * | *** | 6 | |
| Wang Liuqing | 2016 | ** | * | *** | 6 | |
| Seong-Kyu Kim | 2017 | **** | ** | *** | 9 | |
