# Supplementary figures and images for: Expression of interleukin-18 in primary Sjögren syndrome and its potential mechanisms with disease: A systematic review and meta-analysis
Source: Medicine (Baltimore). 2025 Mar 21;104(12):e41919. doi: 10.1097/MD.0000000000041919 (PMC11936574; doi:10.1097/MD.0000000000041919)

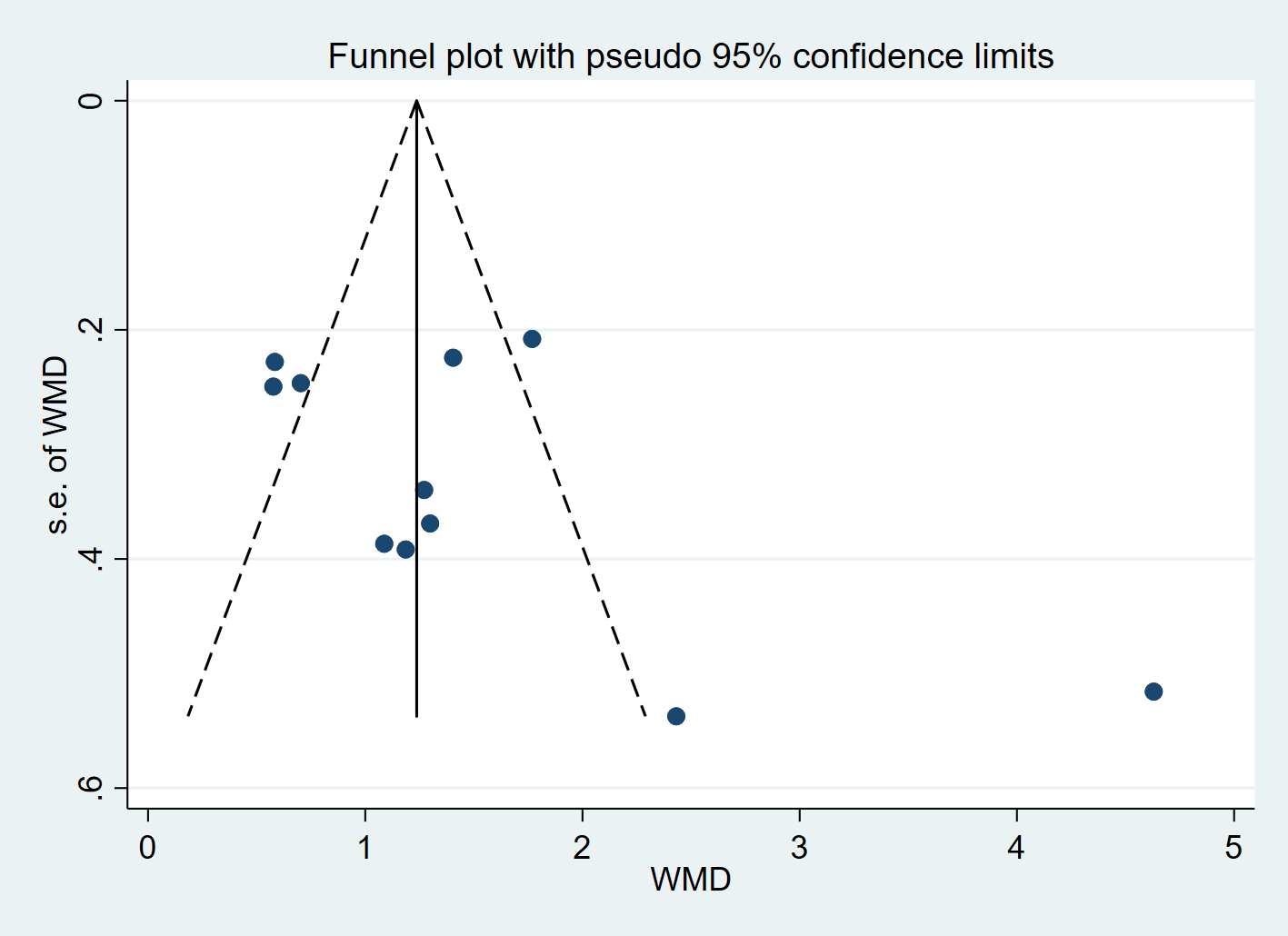

Supplement: SUPPLEMENTARY MATERIAL [file medi-104-e41919-s002.docx]
